# A Study on the Current Situation and Factors Influencing the General Practice Ability of Clinical Undergraduate Students in China

**DOI:** 10.3390/healthcare13070733

**Published:** 2025-03-26

**Authors:** Hai-Jing Ou-Yang, Ying Huang, Lu-Lu Qin

**Affiliations:** Department of Social Medicine and Health Management, School of Public Health, Health Science Center, Hunan Normal University, Changsha 410013, China; 202270193618@hunnu.edu.cn (H.-J.O.-Y.); 202370193595@hunnu.edu.cn (Y.H.)

**Keywords:** general practice, general practice ability, clinical undergraduate students, scale development

## Abstract

Objective: General practice (GP) plays an important role in the healthcare of human populations, and Chinese medical schools have paid attention to using education to improve the GP ability of medical students. However, there are still a lack of evaluating tools focusing on the general practice ability of clinical undergraduate students in China. To the best of our knowledge, this is one of the first studies aimed at constructing a scale of the general practice ability of medical undergraduates (GPA-MU) and understanding the current situation and factors influencing the general practice ability of the undergraduate clinical medical students in China. Methods: This study included two steps: the scale development and the empirical study. The items of the GPA-MU scales were developed from a literature review, in-depth individual interviews, Delphi expert consultation, and a pilot test. For the empirical study, a sample of 318 medical undergraduates was recruited. Item analysis, content validity, exploratory and confirmatory factor analysis, convergent and discriminant validity, and internal consistency reliability were conducted to examine the properties of the GPA-MU, and single and multi-factor analysis were used to explore the related factors of the GPA-MU. Results: Finally, the GPA-MU consisted of 3 first grade indexes, 12 second grade indexes, and 43 third grade indexes, with good reliability and validity, which could be used to assess the level of general practice ability of clinical undergraduate students. According to the cross-sectional study, the general practice ability score of clinical undergraduate students in Hunan Province was 83.75 ± 14.97, indicating the low general practice ability of clinical undergraduate students. The region, knowledge, duration of practice, and salary package had an obvious effect on the general practice ability of clinical undergraduate students. Further targeted intervention strategies in China are needed to strength the general practice ability of students.

## 1. Introduction

General practice (GP), also known as general medicine, originated in North America in the 18th century. Until the 1960s, the American Board of Family Practice (ABFP) was established, officially recognizing GP as a medical specialty in the United States [1]. General practitioners, being the primary providers of medical services, play a crucial role in preventing and controlling diseases, including major epidemics [2]. In China, GP education was introduced over 30 years ago. Undergraduate medical education in China usually lasts for 5 years, including basic medical science courses and clinical practice courses, and after graduation, you need to pass the licensing examination before applying for clinical work. While progress has been made in establishing education and service systems for general practice, there are still significant issues to be explored, particularly in the lack of effective training and selection mechanisms for general practice professionals in the community [1]. In 2011, the State Council of China issued the Guiding Opinions on Establishing a General Practitioner System, aiming to have two to three qualified general practitioners for every 10,000 urban and rural residents by 2020 [3]. In response to this guideline and the need for community-based medical services, China has been actively promoting a grassroots diagnosis model that places emphasis on community prevention and control [4]. Furthermore, these were further emphasized by the National Health Commission of China, who presented the need to strengthen community prevention and control of the novel coronavirus pneumonia epidemic on 24 January 2020 [5,6]. Given these circumstances, there is an urgent need to train competent general practitioners who possess comprehensive skills, can adapt to societal demands, and demonstrate professional abilities.

At present, in most medical colleges, problems such as the relatively weak cultivation of and investment in general practice education for medical undergraduates and the insufficient understanding of general practice among medical students are quite prominent [7]. There are relatively few research achievements in the assessment and evaluation of general practice education for medical undergraduates. Since there is no unified standard for the assessment of general practice education, there is no unified criterion for evaluating the effectiveness of general practice education [8,9,10,11]. Meanwhile, there is a significant gap in the level of medical technology among different countries, and the cultivation models for general practitioners vary from country to country [12,13,14]. This has resulted in a certain gap between the current situation of general practice education in domestic universities and the internationally advanced concepts [15,16]. Only by adopting a scientific attitude and improving the teaching quality control system can the sustainable development of general practice teaching be ensured. Therefore, exploring and constructing a set of evaluation index systems for assessing the general practice ability of medical undergraduates from the perspective of global health is of great academic value and social significance [17,18,19].

This study aims to develop an evaluation index system for general practice ability among medical undergraduates, taking into account the previous research and adapting it to China’s actual circumstances, as well as taking into account global social and economic development and major health issues. This study will also conduct an empirical investigation to assess the level of general practice ability and identify its influencing factors among clinical medical students in Hunan Province, so as to provide a reference for the development of general practice ability in China and other areas. To the best of our knowledge, few studies have developed an evaluation index system for assessing general practice ability among medical undergraduates.

## 2. Materials and Methods

### 2.1. Study Design

This study is a qualitative and cross-sectional survey empirical study conducted in China and is the key project of the National Medical Professional Degree Graduate Education Steering Committee (A-YXGP20210101-01). The qualitative research includes the Delphi expert method and the semi-structured interview method. In addition, a multi-stage random cluster sampling method was adopted to select four medical colleges in Hunan Province. A total of 636 medical students were sampled in the cross-sectional survey to conduct empirical research.

### 2.2. Participants and Procedures

#### 2.2.1. Expert Selection for the Delphi Method

During the period spanning from May to September 2022, a total of 15 experts were carefully selected to participate in this study by using purposive sampling. These experts were chosen based on their expertise in various fields related to general practitioners, including general medical education, general medical research, general practitioner management, and other relevant industries.

The criteria for expert inclusion in this study were as follows: (1) voluntarily participating and being interested in the research content; (2) working in general practice, general medical education, general medical research, general practitioner management, and other related industries for 5 years or more; (3) having a bachelor’s degree or above; and (4) having participated in the formulation of medical-related indicators at the national or provincial ministerial level.

#### 2.2.2. The Subjects of Semi-Structured Interview

This study employed a purposive sampling method to select appropriate participants for the semi-structured interview. From June to August 2022, a range of healthcare facilities, including hospitals at various levels, health administration departments, community health institutions, and individuals with backgrounds in clinical medicine and preventive medicine, were chosen as interview subjects. Group interviews and telephone interviews were conducted to gather data. Initial contact was made by the research team via email and telephone, and the respondents were sent information on the purpose of the study and an informed consent form. Group and telephone interviews were used to collect data. Written informed consent was obtained from respondents for all interviews, which were recorded by trained researchers using digital recording equipment, and transcription of the audio recordings was completed within 24 h of the interviews. The sample size was determined by the principle of information saturation, resulting in a total of 20 subjects being interviewed for this project.

#### 2.2.3. The Subjects of the Reliability and Validity Analysis of the Questionnaire and the Empirical Research

The inclusion criteria were as follows: (1) clinical medical undergraduates; (2) in the fourth or fifth year of college, having successfully completed courses pertaining to general practice, as well as having completed community practice in this field; and (3) willing to participate in this survey and able to understand and complete the survey content. The exclusion criteria were as follows: (1) unable to collaborate with the general practice ability assessment; and (2) unable to participate due to other reasons.

In the reliability and validity analysis of the questionnaire, the method of purposive sampling was adopted in this survey, including upper division students in clinical medicine and preventive medicine programs. Four classes from four medical colleges in Hunan province were selected as the investigation sites. The sample size was calculated to be 5~10 times the number of items, so a sample size of 220 cases was proposed for this survey. However, considering the potential absence of survey objects and invalid questionnaires during the investigation, the sample size was expanded by 10% to ensure an adequate representation. Therefore, the final required sample size for this survey was determined to be 242 cases.

The empirical research conducted in this study employed a multi-stage random cluster sampling method to select the objects. Specifically, the simple random sampling method was used to select four medical schools in Hunan Province as investigation sites (4/10). According to their grades, one class was selected using pure random sampling in the senior and fifth grade, and all the students in the selected class were included in the study using cluster sampling. The cross-sectional study formula was used to calculate the sample size: *N =* $\frac{Z_{1}^{2}-\frac{\alpha }{2}}{\sigma^{2}}$ (1 − *p*) *p*, where $Z_{1}-\frac{\alpha }{2}$ is the percentile corresponding to the area of 1−α/2 under the standard normal distribution, *p* represents the expected incidence, and *σ* is the allowable error [20,21]. According to the pre-survey, 55.2% of the clinical medical students had a sufficient general medicine ability, *α* = 0.05, *δ* = 0.1 *p*. According to the sample size calculation formula, it can be calculated that the required sample size is 310. Expanding this sample size by 10% results in a final required sample size of 341.

### 2.3. Research Methods

#### 2.3.1. Literature Review Method

The search was conducted using various database resources such as CNKI, Wanfang, Weipu, PubMed, and Web of Science. The keywords “general practice”, “general practitioner”, and “family physician” were used to find relevant training programs, assessment programs, policy documents, and related literature on general practice for medical undergraduates in the past ten years and to conduct a systematic review. These resources were obtained from medical colleges, general practice education associations, and education supervision or evaluation institutions, both domestically and internationally. The collected data were analyzed, and as a result, a preliminary theoretical framework of the general practice ability evaluation index system for medical undergraduates was established. This framework consisted of five categories and twenty indexes.

#### 2.3.2. Delphi Method

The Delphi method was employed to conduct two rounds of consultation with a panel of 15 experts. The preliminary phase of expert consultation encompassed the incorporation of diverse communication channels, such as face-to-face interactions, electronic mail, WeChat, and additional written questionnaires.

The expert consultation letter comprised three main sections: (1) a cover letter, which provided an overview of the background, purpose, and significance of the consultation; (2) the expert’s personal information, such as gender, education, professional background, job title, and years of experience, as well as their familiarity with general medicine; and (3) an explanation of how the experts would be expected to complete the form, including instructions for filling in the required information, and a description of the criteria used to assign values to each index. Additionally, the expert letter included a comment form for evaluating the general medicine competency index system.

After four weeks, the questionnaire was collected, and the data were analyzed and organized. The results of the inquiry were formulated based on the feedback received and previous research. During the process of organizing the data, the familiarity level of the experts and the criteria for judgment were reassessed. The modifications suggested by the experts were summarized, and the second round of inquiry questionnaire was developed after revising and improving the indicators. The first round of inquiry and the revised inquiry materials were then provided to the relevant experts. When distributing the second round of inquiry questionnaires, some information from the previous round of inquiries was also shared with the experts to provide a further reference for their judgment.

#### 2.3.3. Semi-Structured Interview Method

Using the method of purposive sampling, from June to August 2022, relevant personnel such as those from hospitals at all levels, health administrative management departments, community health institutions, and previous graduates majoring in clinical medicine and preventive medicine were selected as the interviewees. Interviews were conducted through methods such as group interviews and telephone interviews. The sample size of the interviews in this study was determined according to the principle of information saturation. Eventually, a total of 20 interviewees were interviewed for this project. Before the interview, the research team provided a comprehensive explanation of the purpose, methods, and content of the investigation to the interviewees. With the participants’ informed consent, appropriate locations and times were chosen for the interviews, which followed an open-ended question and answer format. Before collecting and organizing the data, interviews were conducted with the participants and were recorded on site. On average, each interview lasted between 1 and 1.5 h per person. The interview tool utilized in this study consisted of three components: (1) an informed consent form, (2) basic information, (3) an interview outline. Throughout the interviews, great care was taken to protect the privacy of the research subjects and to encourage them to freely express their genuine emotions. The sequencing of the questions could be adjusted to accommodate the interviewees’ preferences and foster more in-depth discussions. Additionally, since the interviewers were meeting the research subjects for the first time, a thorough understanding of both the research subjects and the interview materials was essential in order to facilitate effective communication. Within 48 h following the interviews, a team of two individuals conducted data analysis and organization, assigning participant codes ranging from A1 to A20, and Colaizzi’s seven-step analysis method was used for the analysis of the results.

#### 2.3.4. Questionnaire Survey

In this study, electronic questionnaires and paper questionnaires were distributed to conduct the investigation. The survey tools included socio-demographic information and the Questionnaire for General Practice Ability Assessment of Medical Undergraduates constructed in this project.

The socio-demographic information collected in this study included gender, college, age, the place of origin of the students (urban vs. rural), grade, whether students had signed clinical work agreements (yes or no), whether students’ college entrance examination major was a clinical major or not (yes or no), the degree of understanding about general practitioners, internship time, salary and wages, professional identity, professional prospects, and professional choice.

The questionnaire was divided into three dimensions and 43 items. The three dimensions are as follows: general practice service ability, public health service ability, and knowledge absorption ability. Within general practice service ability, “Final examination score of Introduction to General Practice” was its evaluation item, which was divided into five degrees according to its scores. This item was scored by a Likert-5 rating system; the higher the score, the stronger the general practice ability of the student. In public health service ability, “Community internship evaluation results” were also evaluated, and the score was 1–3 points according to the corresponding level. The other items were self-rated items, which were selected by the respondents according to their self-assessed level of ability. The scoring method involved three levels (3 = have the ability; 2 = partially have; 1 = do not have). The total score was the sum of the scores in each dimension, and the score ranged from 43 to 131 points. The higher the total score and sub-scale score, the better the general practice ability.

### 2.4. Ethical Considerations

This is the key project of the National Medical Professional Degree Graduate Education Steering Committee (A-YXGP20210101-01). In this study, the selected research subjects were informed about the requirement of obtaining informed consent, and the data of the participants were kept confidential in strict accordance with the relevant professional ethical standards.

### 2.5. Statistical Analysis

In this study, the statistical software SPSS 24.0 (IBM, Armonk, NY, USA) was utilized to input the data and perform the necessary statistical analysis. The AMOS 23.0 (IBM, Armonk, NY, USA) software was utilized to conduct a confirmatory factor analysis on the first-order indicators of the scale, A1, A2, and A3, in order to validate the second-order model. Descriptive statistics, such as the average, standard deviation, frequency, rate, and composition ratio, were employed to depict the demographic characteristics and general practice of the participants. To compare the disparity in general practice ability among clinical medical undergraduates with distinct characteristics, the *U*-test and *H*-test were employed. Additionally, unconditioned binary logistic regression analysis was conducted to explore the factors that influence the general practice ability of clinical medical undergraduates. It is important to note that all statistical analyses in this paper were conducted using bilateral tests, with a significance level of α = 0.05.

## 3. Results

### 3.1. The Results of the Literature Analysis

A total of 1586 references were initially retrieved, and after carefully reviewing the titles and abstracts, 1461 were excluded. Additionally, 10 duplicate articles and 62 irrelevant articles were removed from the study. Eventually, 53 articles remained. By analyzing these references, the researchers combined the Accreditation Council on Graduate Medical Education (ACGME) ability assessment with the three-orientation ability assessment of clinicians in China. As a result, they developed an initial theoretical framework for evaluating the general practice ability of medical undergraduates.

This framework encompassed five categories and 20 indexes, including general practice service delivery ability (responsibility, compassion, resilience, clinical practice ability, information gathering ability, decision-making ability), health advocacy and promotion ability (affinity, communication ability, network building ability, ability to cultivate others), interpersonal communication and leadership ability (presentation ability, teamwork ability, team building ability, team leadership), safety assurance and quality improvement ability (safe operation ability, quality management ability, self-control ability, etc.), and research, teaching, and self-improvement ability (professional learning ability, innovation ability, development of others).

### 3.2. The Results of the Semi-Structured Interviews

#### 3.2.1. General Information of Expert

Ultimately, interviews were conducted with 20 individuals, who were assigned the following codes: A1, A2, A3, A4, A5, A6, A7, A8, A9, A10, A11, A12, A13, A14, A15, A16, A17, A18, A19, and A20. This sample consisted of two administrative personnel in the general medical departments of health-related agencies, five teachers from higher medical institutions, six directors of township health centers, five general practitioners in rural areas, and two clinical physicians from tertiary hospitals.

#### 3.2.2. Topic Extraction

This study employed interview data and literature analysis to extract and analyze a primary item pool of three main themes: general practice diagnostic and treatment service ability, public health service ability, and knowledge absorption ability. The preliminary frameworks were constructed for the three main themes, considering their conceptual connotations, constituent dimensions, and respective weights. Finally, the primary item pool was then organized into a draft expert inquiry questionnaire, which consisted of three main themes, 12 secondary items, and 47 tertiary items. The three main themes were as follows:

Theme 1: General Practice Diagnostic and Treatment Service Ability

The surveyed respondents generally believed that general practitioners play the role of a gatekeeper in primary healthcare. They should possess solid clinical theoretical knowledge and proficient clinical operation skills. A19: Solid theoretical knowledge and proficient clinical operation skills are the most basic ability requirements for doctors. Without solid theoretical knowledge and proficient clinical operation skills, one cannot even pass the licensing examination for doctors, let alone serve the majority of people. A15: Only with proficient theoretical knowledge can a doctor make a clinical diagnosis of patients based on clinical symptoms and examination results, and carry out targeted drug treatment for patients according to corresponding pharmacological knowledge. A4: General practitioners serve at the grassroots level. Their main purpose is to conduct preliminary diagnosis of patients, and treat diseases with mild symptoms and those that are relatively easy to treat. For complex diseases, they should be able to distinguish and refer patients to corresponding specialized hospitals. Therefore, general practitioners need to have comprehensive clinical diagnosis and treatment capabilities. They don’t have to be highly specialized, but they must have a wide range of knowledge. A6: As front-line doctors at the grassroots level, general practitioners interact with the general public the most. They should have good professional ethics and communication skills. With the increasing aging of the population, they should also possess certain end-of-life care capabilities.

Theme 2: Public Health Service Ability

Most interviewees believed that, as a general practitioner, in addition to having basic diagnostic and treatment capabilities, one should also possess public health service capabilities. A3: Currently, most of the work of general practitioners at the grassroots level is mainly responsible for the treatment and control of chronic non-communicable diseases. For the treatment and prevention of chronic diseases, general practitioners are required to have good public health qualities and be able to understand the risk factors and prevention and control measures of various chronic non-communicable diseases. A5: After the outbreak of the COVID-19 pandemic, the focus of general practitioners’ work shifted to public health work such as the prevention and control of infectious diseases and health education. After the subsequent changes in the epidemic prevention policies, general practitioners should possess strong public health service capabilities to prevent the resurgence of COVID-19. A17: After the signing of the family doctor service contract, each resident has his/her own health record. General practitioners should learn to use the health records to monitor the health conditions of each resident and be able to provide timely diagnosis and treatment and targeted health services to patients.

Theme 3: Knowledge Absorption Ability

The interviewees unanimously believed that, as a student, only by digesting and absorbing knowledge and transforming it into one’s own ability can learning be effective and can one participate in the diagnostic and treatment services of general practitioners more quickly. A18: Nowadays, college students are educated in a cramming way. How much of what students learn in class can be transformed into their own knowledge determines their ability level as general practitioners. Only when all knowledge is absorbed and transformed into one’s own can one reach the level of a qualified general practitioner. A20: Learning must keep pace with the times. Now that scientific development is changing very rapidly, one must keep up with the development of society. Also, only with a clear plan can the learning efficiency be improved.

### 3.3. Results of the Expert Consultation

#### 3.3.1. General Information of the Experts

In this research, a series of two rounds of communication with experts was carried out via email with the same panel of experts. A total of 15 experts were involved in the study, with 53.3% of them being male and 46.7% female. In terms of their educational background, 53.3% held a doctorate degree while 33.3% held a master’s degree. Furthermore, eight experts held senior professional titles, which accounted for 53.3% of the total. The participating experts represented various fields, including grass-roots health management, general practice, general practice education, health policy, and medical teaching, among others. It is worth noting that 66.7% of the experts had work experience of 16 years or more. Details are in Table 1.

#### 3.3.2. The Level of Expertise, Degree of Opinion Coordination, and Level of Authority Exhibited by the Experts

The recovery rate of the two rounds of expert correspondence questionnaires was 100%, indicating a high level of interest and cooperation from the experts. The total Kendall’s W coefficients for these two rounds of correspondence were 0.518 and 0.630, respectively, demonstrating a high level of consistency in the experts’ responses. This suggests a strong level of agreement among the experts and supports the reliability of the results obtained through the correspondence. The authority coefficient of the two rounds of letter consultation exceeded 0.7, further indicating the reliability of the results obtained through the written correspondence. Details are in Table 2.

#### 3.3.3. The Results of Expert Consultation

(1) The results of the first expert consultation

The importance values of the primary indicators were evenly distributed between 0.73 and 0.92, with a coefficient of variation (CV) ranging from 0.06 to 0.14. The experts expressed a high level of agreement with the primary indicators. The importance values of the secondary indicators ranged from 7.67 to 9.80, with a CV ranging from 0.06 to 0.22. No modifications were suggested by the experts for the secondary indicators. The importance values of the tertiary indicators ranged from 7.47 to 9.67, with a CV from 0.06 to 0.32. Among the tertiary indicators, four indicators, namely “information retrieval ability (C252)”, “innovation ability (C314)”, “clear planning or goals in the field of general practice medicine (C332)”, and “pride in becoming a general practitioner (C333)” had coefficients of variation greater than 0.25 and were therefore removed. In terms of operational assignment scores, the values ranged from 7.87 to 9.40, with a coefficient of variation ranging from 0.1 to 0.38. Among the tertiary indicators, a total of 13 indicators (C143, C213, C222, C223, C224, C225, C231, C243, C253, C254, C321, C322, and C331) had CVs greater than 0.25, and modifications were made to their assignment values based on expert opinions.

(2) The results of the second expert consultation

The results of the second round of inquiry indicated that the importance values and CVs of the retained indicators from the first round were consistent with the screening system constructed in this study. Through reliability and validity analysis of the questionnaire data, as well as factor analysis, it was found that the evaluation indicator system of the comprehensive medical ability of medical undergraduates constructed in this study had high reliability and applicability. Thus, an evaluation indicator system for the comprehensive medical ability of medical undergraduates has finally been formed in this study, consisting of 58 indicators divided into three levels, including 3 primary indicators (comprehensive diagnosis and treatment service ability, public health service ability, and knowledge absorption ability), 12 secondary indicators, and 43 tertiary indicators. According to the analysis of the weight coefficients assigned to each indicator by experts and the feedback from the questionnaire, the importance values and coefficient of variation in each primary, secondary, and tertiary indicator were determined. The mean importance value of the primary indicators ranged from 8.87 to 9.60, with a CV ranging from 0.08 to 0.19; the mean importance value of the secondary indicators ranged from 7.67 to 9.80, with a CV ranging from 0.04 to 0.23; and the importance values of tertiary indicators ranged from 8.40 to 9.60, with a CV ranging from 0.06 to 0.25. The mean operability value of the tertiary indicators ranged from 8.47 to 9.73, with a CV ranging from 0.08 to 0.23.

#### 3.3.4. The Outcome of the Weightings of GPA-MU

(1) The maximum eigenvalue (λ_max_) and consistency results

The overall consistency ratio (CR) of the study and the CR between the primary and secondary indicators, both being less than 0.01, indicated that the constructed matrix of indicators satisfied the criteria of consistency. As a result, the weight vector could be utilized in decision analysis applications.

(2) The outcome of weights and combination weights

In this study, the primary indicators were as follows: general practice diagnostic and treatment service ability (0.344), public health service ability (0.339), and knowledge absorption ability (0.317). Among the secondary indicators, the top three weighted coefficients were cognitive implicit knowledge stock (0.340), emotional implicit knowledge stock (0.334), and belief implicit knowledge stock (0.325), while the lowest weighted coefficient was the capability of managing mental illnesses, which was only 0.202. Among the tertiary indicators, the top two weighted coefficients were the ability to master and adhere to physician professional ethics, ethical principles, and protect patient privacy (0.344), and the ability to empathize with patients (0.344). The top four combined weighted coefficients were problem-solving capability (0.037), empathy towards patients (0.036), emotional self-control (0.036), and the ability to absorb new knowledge (0.036).

### 3.4. Reliability and Validity Analysis of the GPA-MU

(1) General information of subjects

A total of 242 questionnaires were distributed in this survey, and 222 valid questionnaires were ultimately collected, resulting in a questionnaire recovery rate of 91.7%. Among the respondents, males accounted for 46.4% and females accounted for 53.6%. A total of 64.9% of the students surveyed were from rural areas. Fourth-year students accounted for 36.5%, while students who had signed clinical work agreements accounted for 24.3%. Furthermore, 85.6% of the students indicated that clinical medicine was their first choice for the college entrance examination. Details are in Table 3.

(2) Reliability test of the GPA-MU

The results of the Cronbach’s α coefficient for each index are presented in Table 4. The overall index system demonstrated a Cronbach’s α coefficient of 0.983, while the primary index displayed coefficients of 0.944, 0.980, and 0.928, respectively. The secondary indexes exhibited a range of Cronbach’s α coefficients from 0.821 to 0.982, indicating a high level of internal consistency within the scale.

(3) Validity test of the GPA-MU

The KMO statistic yielded a value of 0.944, while the Bartlett sphericity test resulted in a *p*-value less than 0.001 (*df* = 903). These findings suggest that the indicators used in the analysis were not independent, but rather exhibited correlation, indicating their suitability for factor analysis. Details are in Table 5.

The results of the exploratory factor analysis are presented in Table 6. For the orthogonal rotation factor analysis, the common factor was set at a fixed value of 3. The analysis revealed that the characteristic roots of the seven factors exceeded 1, indicating their significance. The cumulative variance contribution rate was found to be 71.752. However, based on the previous theoretical analysis, the factors were further categorized into three groups. The cumulative variance contribution rate for these three factors was 62.994%, which can be considered to be a high level of variance contribution. By examining the factor load matrix for each project (Table 7), it was observed that index A1 had a factor load > 0.4, primarily concentrated in factor 3; index A2 had a factor load > 0.4, primarily concentrated in factor 1; and index A3 had a factor load > 0.4, primarily concentrated in factor 2. This indicates that factor 3 represents general practice diagnosis and treatment ability, factor 1 represents public health service ability, and factor 2 represents knowledge absorption ability. These findings align with the structure of the evaluation index system for assessing general practice ability among medical undergraduates.

Table 8 shows the results of confirmatory factor analysis. In the current study, the software AMOS23.0 was used to conduct a confirmatory factor analysis of the second-order model for A1, A2, and A3. Table 9 showed that the goodness of fit index (GFI) of the model was 0.961, the comparative fitness index (CFI) was 0.956, and the benchmark fitness index (NFI) was 0.945, all of which were above 0.9. The Root Mean Square Error of Approximation (RMESE) was 0.072, less than 0.1, indicating that the overall fit of the model with a well-fitting confirmatory analysis model was better. Since there were multidimensional scales in the model, these scales needed to be analyzed separately, and the results showed that the model fit of each dimension scale was good. The factor load of each item ranged from 0.611 to 0.909; the AVE value of each latent variable ranged from 0.659 to 0.0.691, all greater than 0.65; and the CR value of each latent variable ranged from 0.899 to 0.973, all close to or greater than 0.9, indicating that the model had good aggregation validity.

### 3.5. Current Situation of General Practice Ability of Clinical Medical Undergraduates in Hunan Province

#### 3.5.1. The Socio-Demographic Information of the Participants

The findings regarding the socio-demographic information are presented in Table 10. A total of 341 questionnaires were distributed, out of which 318 were considered valid, resulting in an effective recovery rate of 93.25%. Among the 318 medical students, 54.40% were female, 65.1% hailed from rural areas, and 72 were rural-oriented clinical medicine students, accounting for 22.6% of the total. Additionally, 85.2% of students had selected clinical medicine as their first choice in the college entrance examination, while 80.8% expressed no intention of becoming a general practitioner in the future.

#### 3.5.2. General Practice Ability Score of Clinical Medical Undergraduates in Hunan Province

The findings of this study revealed that the overall general practice ability score of clinical medicine undergraduates was 83.75 ± 14.97. Specifically, the score for general practice service ability was 32.60 ± 5.64, the score for public health serviceability was 37.82 ± 7.59, and the score for knowledge absorption ability was 13.33 ± 2.57. Details are in Table 11.

### 3.6. Analysis of Influencing Factors on the Current Situation of Clinical Medical Undergraduates’ General Practice Ability in Hunan Province

#### 3.6.1. Single Factor Analysis

The results of single factor analysis are shown in Table 12. The place of origin of the students, the degree of understanding about general practitioners, internship time, salary and wage, sense of identity, employment prospects, and inclination towards becoming a general practitioner upon graduation were the factors influencing the general practice ability of the medical undergraduates.

#### 3.6.2. Multivariate Analysis

The study findings indicated that the city of origin (OR = 0.403, 95%CI: 0.236–0.688) along with a higher level of knowledge about general practitioners (OR = 0.011, 95%CI: 0.001–0.125) were identified as independent harmful factors for general practice ability. Conversely, a longer internship time (OR = 3.058, 95%CI: 1.478–6.327), a high salary and wage (OR = 2.486, 95%CI: 1.220–5.068), and a high professional identity, (OR = 2.496, 95%CI: 1.193–5.222) were determined to be independent predictors of general practice ability. Details are in Table 13 and Table 14.

## 4. Discussion

### 4.1. The Scientific Rigor of the Conducted GPA-MU Scale

Based on the feedback from two rounds of expert consultations, the response rates for both rounds of questionnaires reached 100.0%. This indicated the high level of attention and cooperation from the experts towards this study. According to the relevant research [22], an authority coefficient ≥ 0.70 indicates a good level of authority for Delphi expert consultations. In this study, all expert authority coefficients exceeded 0.70, indicating that the expert consultations in this study were authoritative and trustworthy. The Kendall’s W coefficients for the two rounds of consultations were 0.518 and 0.630, respectively. According to research [23], the range of values for the coordination coefficient is 0–1, with larger values indicating better coordination. Therefore, the high level of agreement among expert opinions in these two rounds indicated a good level of coordination and a high consistency of expert opinions. The coefficient of variation for all the indicators in the final indicator system formed in this study was less than 0.25, indicating good coordination and consistency among the expert opinions on the questionnaire constructed for this study. This high acceptance of the comprehensive medical competency evaluation indicator system constructed in this study demonstrated the reliability of the expert consultation results.

Based on the calculation results of the indicator weights, the CR values of each indicator were all less than 0.01. According to relevant studies, matrix A is considered to have satisfactory consistency when the CR is less than 0.1 or λ_max_ = n and *CI* = 0. Therefore, it can be concluded that the constructed indicator system matrix met the requirements of consistency indicators, and the weight vector could be used for decision analysis applications [24].

The results of the Analytic Hierarchy Process show that in the evaluation indicator system of the general medical competency of undergraduate clinical medical students, 3 primary indicators (with weights of 0.344, 0.339, and 0.317, respectively), as well as 12 secondary indicators and 43 tertiary indicators, had weights that could be used for comparative analysis among the indicators.

The final grades of responses regarding general medicine courses and internship evaluations were considered as evaluation criteria in the tertiary indicators, which had collaborative aspects with previous studies conducted by experts [25]. Furthermore, from a holistic perspective, this indicator system not only aligned with the development trend of general medicine domestically and internationally but also possessed specificity to general medical students. Therefore, the findings of this study can to some extent provide a certain reference basis for the future cultivation of general medical talents in our country. On one hand, in terms of content, it not only satisfied the current needs of general physician training in our country, but it also collaborated with the comprehensive quality evaluation indicator system for general physicians established by Bao Yuhua and others [26,27,28], as well as with the framework of general physician competency established by Fang Jinming et al., and other contents of past research [29,30,31,32,33].

### 4.2. The Reliability and Validity of the GPA-MU Scale

It is widely accepted among scholars both domestically and internationally that a Cronbach’s α coefficient greater than 0.75 indicates a reliable result [34]. In this study, the Cronbach’s α coefficient for the indicator system was found to be 0.983. Furthermore, all three primary indicators had Cronbach’s α coefficients exceeding 0.9, while all twelve secondary indicators had Cronbach’s α coefficients exceeding 0.8. These findings demonstrate that the indicator system exhibited a high level of internal consistency and that the scale’s stability is quite high.

The overall correlation coefficient between the general index system and the three primary indicators was >0.9. The secondary indicators were correlated with the primary indicators, tertiary indicators, and secondary indicators with a correlation coefficient > 0.5, which was statistically significant [35]. This indicated a strong correlation between each indicator and the higher level indicator, suggesting that the homogeneity of each item was better than that of the overall scale [36]. By using a comprehensive evaluation model based on factor analysis which incorporated the overall index system of the high and low score groups, it was evident that this was an effective comprehensive assessment system [37]. Clearly, this index system could effectively distinguish medical undergraduate students at different levels of general medical ability.

By employing exploratory factor analysis, three common factors were extracted, with a cumulative variance contribution rate of 62.994%. This indicated that the data information within the indicator system accounted for 62.994% of the total, implying that the structure and characteristics of the indicator system could be reasonably explained and could objectively reflect the general medical capabilities of undergraduate medical students. The results of confirmatory factor analysis showed that the fit indexes of the model were as follows: the goodness-of-fit index (GFI) was 0.961, the comparative fit index (CFI) was 0.956, and the normed fit index (NFI) was 0.945, all exceeding 0.9. The Root Mean Square Error of Approximation (RMSEA) was 0.072, which was less than 0.1, indicating a good fit of the confirmatory analysis model and a good overall model fit [38]. Moreover, the fit of the dimensional scale models in this model were also good. The factor loadings of each item ranged from 0.611 to 0.909, and the average variance extracted (AVE) values of each latent variable ranged from 0.659 to 0.691, all exceeding 0.65. The composite reliability (CR) values of each latent variable ranged from 0.899 to 0.973, all approaching or exceeding 0.9, indicating the good convergent validity of the model. In conclusion, the validity of the indicator system constructed in this study to evaluate the general medical capabilities of undergraduate medical students was good.

### 4.3. General Medicine Ability Level of Clinical Medical Undergraduates in Hunan Province

Of the 318 medical students included in the study, 80.8% would not choose to become a general practitioner in the future. The general practice ability score of the medical undergraduates was 83.75 ± 14.97 points, among which the general practice service ability score was 32.60 ± 5.64 points, the public health service ability score was 37.82 ± 7.59 points, and the knowledge absorption ability score was 13.33 ± 2.57 points. Except for the general practice service ability, the other abilities were lower than the corresponding median score, indicating that the general practice ability of clinical medical students was still at a poor level at this stage, and the expected effects of training had not been achieved. The results of this study are consistent with those of Bo Hong [39] and Zhang Huaibin [40]. This may be because undergraduate medical education in China is universal education, and students’ definition of general practitioners is limited to the level of awareness that doctors only need to have excellent professional knowledge and professional skills. Their definition does not take into account the improvement of one’s professional communication ability, information processing ability, public health service ability, and knowledge absorption ability. Therefore, in addition to the general practice service ability, the other abilities were at a low level. Studies have shown [41] that with an increase in the years engaged in general practice, the competency of general practitioners has further increased. The objects of this study were medical students studying for four or five years who had undergone clinical practice but had not yet undergone systematic standardized training. Medicine is an empirical subject, and medical students have not systematically practiced medicine; their ability to turn theoretical knowledge into practical ability is limited, resulting in their current general medicine ability level being low. With the development of society, the general public’s demand for general practitioners to have a high level of is no longer limited to their use of technology, and the need for the humanistic quality of doctors is also increasing. Therefore, it is suggested that medical colleges should also pay attention to improving clinical medical students’ professional spirit, concept of great health, and ability to transform and absorb knowledge in teaching and training.

### 4.4. Influencing Factors of Clinical Medical Undergraduates’ General Practice Ability Level in Hunan Province

The study indicated that the place of origin of the students was primarily urban and that the higher degree of understanding about general practitioners was an independent detrimental factor to the ability of general medical practitioners. This research result is consistent with that of Le Floc’h B [42]. One possible explanation for this correlation is that general practitioners in China primarily serve at the grassroots level, where the workload is heavier, and the income is not comparable to that of specialist doctors in top-tier hospitals. As a result, clinical medical students have a cognitive bias towards the profession of general practitioners. The more they are aware of the current employment situation for general practitioners, the less inclined they are to pursue this career path, and the less they prioritize improving their general medical skills. In terms of the regional source of students, medical students from rural areas may care more about a stable paid job due to family reasons. Comparing the employment pressures, capital, and time investments of clinical specialists, medical students from rural areas are more inclined to serve as general practitioners at the grassroots level with less employment pressure.

A long probation time, high salary, and the high professional identity of general practitioners were independent favorable factors for the general practice ability of clinical medical students. This research result is consistent with that of Fang [43]. It is suggested that during internships or practice, medical students are able to effectively apply the knowledge acquired from textbooks, thereby developing relevant skills. This ability continues to evolve with the duration of the internship. Furthermore, a higher salary and a stronger sense of professional identity are more appealing to students [44]. As indicated by Deutsch [45], the salary associated with a particular career choice significantly influences students’ selection of a major. In the 1960s, the social exchange theory was proposed, stating that individuals are more likely to adopt behaviors that they perceive as valuable. Therefore, if the profession of general practitioner is deemed as valuable to medical undergraduates, such as by offering a salary that meets their material needs or a status that fulfills their spiritual aspirations, clinical medical students will be more inclined to actively enhance their general medicine-related abilities during their undergraduate studies.

## 5. Strengths and Limitations

As far as we know, this is the first study to develop an evaluation index system for general practice ability among medical undergraduates. This study developed the GPA-MU scale for the evaluation of medical undergraduate education in China. Taking into account China’s unique circumstances and global socioeconomic development, as well as the main health challenges, this research endeavor sought to provide research tools and reference materials to support the reform and improvement of comprehensive medical education for undergraduate medical students in China. A scholarly investigation was conducted to empirically study the relevant factors that influence the comprehensive medical capabilities of undergraduate medical students. In summary, it is suggested that medical colleges should not only optimize and reform the teaching methods, but also pay attention to improving the internship time, correcting the cognitive bias of clinical medical undergraduates towards general practice, and improving the subjective initiative of medical undergraduates in general practice education.

However, there are some limitations to this study, and our results should be interpreted with caution. The questionnaire survey was limited to medical schools in Hunan Province. Therefore, in the future, it will be necessary to further expand the sample size and survey scope. Additionally, the collected questionnaires were self-completed, which may have led to measurement bias affecting the research results. In the future, we will continue to investigate the current situation and factors influencing general practice competency among general medical students in different regions.

## 6. Conclusions

This study has developed the GPA-MU scale for the evaluation of medical undergraduate general practice ability in China, which was found to have reliability and validity. After assessing the current situation of the general medicine ability of clinical medical undergraduates in Hunan province and exploring its related influencing factors, we found that the city of origin and the high degree of understanding of general practitioners were independent harmful factors for general practice ability, while the long internship, high salary, and professional identity of general practitioners were independent favorable factors for general practice ability. The results of this study could provide a reference for the formulation of relevant policies and for the education of clinical medical undergraduates and could also point out the direction for the training of general practitioners.

## Figures and Tables

**Table 1:** The general information of the experts.

Variables	Classifications	*n* (%)
Gender	Male	8 (53.3%)
	Female	7 (46.7%)
Academic qualifications	Bachelor’s degree	2 (13.3%)
	Master’s degree	5 (33.3%)
	Doctor	8 (53.4%)
Professional title	Entry level	2 (13.3%)
	Intermediate level	5 (33.3%)
	Senior	8 (53.4%)
Years of work experience	5–15	5 (33.3%)
	16–	10 (66.7%)
Research field	Primary healthcare management	2 (13.4%)
	General practice	6 (40.0%)
	General practice education	4 (26.6%)
	Health policies	1 (6.6%)
	Medical education	2 (13.4%)

**Table 2:** The coordination coefficients of the experts and their corresponding verification.

Indicators	The First Round				The Second Round			
	Primary	Secondary	Tertiary	Total	Primary	Secondary	Primary	Total
*Kendall’s W*	0.667	0.488	0.570	0.518	0.667	0.639	0.657	0.630
*df*	28.000	81.933	383.225	456.843	28.023	107.361	405.008	519.980
*v*	2	11	46	61	2	11	42	57
*P*	0.014	<0.001	<0.001	<0.001	0.014	<0.001	<0.001	<0.001

**Table 3:** The general information of the subjects.

Variables	Classifications	Number (*n*)	Percentage (%)
Gender	Male	103	46.4
	Female	119	53.6
The place of origin of the students	Rural	144	64.9
	Urban	78	35.1
Grade	Fourth year	81	36.5
	Fifth year	141	63.5
Students who have signed clinical work agreements	Yes	54	24.3
	No	168	75.7
The major of your college entrance examination is clinical major or not	Yes	190	85.6
	No	32	14.4
The degree of understand about general practitioners	Not really understand	19	8.6
	Don’t understand much	42	18.9
	General understand	123	55.4
	Better understand	30	13.5
	Understand very well	8	3.6
Internship time	1 week and under	52	23.4
	2 weeks	71	32.0
	3 weeks	15	6.8
	4 weeks and above	84	37.8
What do you think of the salary and wages of general practitioners?	Very bad	19	8.6
	Poor	50	22.5
	Average	137	61.7
	High	14	6.3
	Very high	2	0.9
How is your sense of identity for general practitioners?	Lower	9	4.1
	Low	32	14.4
	Average	129	58.1
	Higher	42	18.9
	Very high	10	4.5
What do you think of the employment prospects for general practitioners?	Very bad	7	3.2
	Poor	28	12.6
	Average	109	49.1
	High	65	29.3
	Very high	13	5.9
Will you choose to become a general practitioner after graduation?	Yes	55	24.8
	No	167	75.2

**Table 4:** Internal consistency of the GPA-MU.

Index	Number of Items	Cronbach’s α Coefficient
Total	43	0.977
A1	17	0.921
B11	5	0.740
B12	5	0.808
B13	4	0.883
B14	3	0.813
A2	19	0.973
B21	5	0.894
B22	5	0.888
B23	3	0.891
B24	3	0.905
B25	3	0.918
A3	7	0.896
B31	3	0.899
B32	3	0.802
B33	-	-

**Table 5:** KMO and Bartlett sphericity test of the GPA-MU.

**KMO**		0.944
**Bartlett Spherical Test**	Approximate Chi-square	9798.468
	*df*	903
	*p*-value	0.000

**Table 6:** Variance interpretation rate of the GPA-MU.

Factors	Characteristic Root			Rotational Front Difference Interpretation Rate			Explanation Rate of Variance After Rotation		
	Characteristic Root	Variance Interpretation Rate %	Accumulation%	Characteristic Root	Variance Interpretation Rate %	Accumulation %	Characteristic Root	Variance Interpretation Rate %	Accumulation %
1	22.823	53.076	53.076	22.823	53.076	53.076	12.685	29.501	29.501
2	2.510	5.838	58.914	2.510	5.838	58.914	7.771	18.073	50.703
3	1.754	4.080	62.994	1.754	4.080	62.994	5.285	12.291	62.994
4	1.466	3.409	66.402	-	-	-	-	-	-
5	1.241	2.887	69.289	-	-	-	-	-	-
6	1.059	2.463	71.752	-	-	-	-	-	-
7	1.059	2.463	71.752	-	-	-	-	-	-
8	0.992	2.307	74.059	-	-	-	-	-	-
9	0.751	1.747	77.766	-	-	-	-	-	-
10	0.732	1.702	79.468	-	-	-	-	-	-
11	0.656	1.526	80.994	-	-	-	-	-	-
12	0.631	1.467	82.461	-	-	-	-	-	-
13	0.592	1.378	83.838	-	-	-	-	-	-
14	0.545	1.268	85.107	-	-	-	-	-	-
15	0.534	1.243	86.350	-	-	-	-	-	-
16	0.480	1.115	87.465	-	-	-	-	-	-
17	0.407	0.947	88.412	-	-	-	-	-	-
18	0.402	0.935	89.347	-	-	-	-	-	-
19	0.387	0.900	90.247	-	-	-	-	-	-
20	0.376	0.875	91.122	-	-	-	-	-	-
21	0.355	0.826	91.948	-	-	-	-	-	-
22	0.329	0.765	92.712	-	-	-	-	-	-
23	0.312	0.725	93.437	-	-	-	-	-	-
24	0.291	0.676	94.113	-	-	-	-	-	-
25	0.246	0.571	94.684	-	-	-	-	-	-
26	0.234	0.545	95.229	-	-	-	-	-	-
27	0.210	0.488	95.717	-	-	-	-	-	-
28	0.204	0.473	96.190	-	-	-	-	-	-
29	0.177	0.412	96.602	-	-	-	-	-	-
30	0.170	0.395	96.998	-	-	-	-	-	-
31	0.160	0.372	97.370	-	-	-	-	-	-
32	0.147	0.341	97.711	-	-	-	-	-	-
33	0.136	0.317	98.028	-	-	-	-	-	-
34	0.127	0.295	98.322	-	-	-	-	-	-
35	0.110	0.255	98.577	-	-	-	-	-	-
36	0.106	0.246	98.823	-	-	-	-	-	-
37	0.095	0.221	99.044	-	-	-	-	-	-
38	0.090	0.210	99.254	-	-	-	-	-	-
39	0.085	0.197	99.451	-	-	-	-	-	-
40	0.075	0.173	99.624	-	-	-	-	-	-
41	0.067	0.156	99.781	-	-	-	-	-	-
42	0.054	0.126	99.906	-	-	-	-	-	-
43	0.040	0.094	100.000	-	-	-	-	-	-

**Table 7:** Factor load matrix of the GPA-MU.

Name	Factor Load Matrix		
	Factor 1	Factor 2	Factor 3
C111			0.533
C112			0.629
C113			0.738
C114			0.645
C115			0.695
C121			0.630
C122			0.699
C123			0.662
C124		0.669	
C125			0.597
C131			0.591
C132			0.522
C133			0.673
C134	0.707		
C141			0.692
C142			0.544
C143			0.599
C211	0.511		
C212	0.652		
C213	0.713		
C214	0.664		
C215	0.569		
C221	0.846		
C222	0.719		
C223	0.779		
C224	0.660		
C225		0.406	
C231	0.774		
C232	0.703		
C233	0.783		
C241	0.833		
C242	0.793		
C243	0.807		
C251	0.800		
C252	0.687		
C253	0.796		
C311		0.617	
C312		0.550	
C313		0.426	
C321		0.503	
C322		0.674	
C323		0.571	

**Table 8:** The analysis of the aggregate validity.

Dimension	Item	Factor Loading	CR	AVE
A1	C111	0.618	0.935	0.691
	C112	0.727		
	C113	0.781		
	C114	0.791		
	C115	0.775		
	C121	0.729		
	C122	0.646		
	C123	0.724		
	C124	0.708		
	C125	0.616		
	C131	0.644		
	C132	0.674		
	C133	0.717		
	C134	0.614		
	C141	0.642		
	C142	0.615		
	C143	0.773		
A2	C211	0.716	0.973	0.659
	C212	0.691		
	C213	0.847		
	C214	0.769		
	C215	0.862		
	C221	0.909		
	C222	0.875		
	C223	0.848		
	C224	0.865		
	C225	0.832		
	C231	0.896		
	C232	0.797		
	C233	0.808		
	C241	0.804		
	C242	0.743		
	C243	0.870		
	C251	0.785		
	C252	0.826		
	C253	0.714		
	C311	0.873	0.899	0.667
	C312	0.859		
	C313	0.831		
	C321	0.648		
	C322	0.824		
	C323	0.646		
	C331	0.611		

**Table 9:** Overall fitting coefficient table.

	χ^2^/*df*	RMSEA	NFI	GFI	IFI	CFI
Total scale	2.408	0.072	0.945	0.961	0.976	0.956
A1	2.309	0.092	0.903	0.905	0.941	0.940
A2	2.550	0.083	0.923	0.921	0.950	0.949
A3	2.525	0.084	0.908	0.901	0.922	0.921

**Table 10:** The results of the socio-demographic information.

Variables	Classifications	Number (*n*)	Percentage (%)
Gender	Male	145	45.60
	Female	173	54.40
The place of origin of the students	Rural	207	65.10
	Urban	111	34.90
Grade	Fourth year	118	37.10
	Fifth year	200	62.90
Students who have signed clinical work agreements	Yes	72	22.60
	No	246	77.40
The major of your college entrance examination is clinical major or not	Yes	271	85.20
	No	47	14.80
The degree of understand about general practitioners	Not really understand	26	8.20
	Don’t understand much	63	19.80
	General understand	174	54.70
	Better understand	45	14.20
	understand very well	10	3.10
Internship time	1 week and under	78	24.50
	2 weeks	92	28.90
	3 weeks	21	6.60
	4 weeks and above	127	39.90
What do you think of the salary and wages of general practitioners	Very bad	24	7.50
	Poor	70	22.00
	Average	196	61.60
	High	25	7.90
	Very high	3	0.90
How is your sense of identity for general practitioners?	Lower	63	19.80
	Low	43	13.50
	Average	182	57.20
	Higher	13	19.80
	Very high	17	5.30
What do you think of the employment prospects for general practitioners?	Very bad	9	2.80
	Poor	99	11.60
	Average	153	48.10
	High	36	31.10
	Very high	21	6.60
Will you choose to become a general practitioner after graduation?	Yes	61	19.20
	No	257	80.80

**Table 11:** General practice ability assessment scores of medical undergraduates.

Scores	Maximum	Minimum	$\bar{x}$ ± *s*
Total	43	131	83.75 ± 14.97
General practice service ability	17	53	32.60 ± 5.64
Public health service ability	19	57	37.82 ± 7.59
Knowledge absorption ability	7	21	13.33 ± 2.57

**Table 12:** Single factor analysis of general practice ability of clinical medical students.

Variables	Classifications	*M* (*P*_25_, *P*_75_)	*Z/H*	*p*
Gender	Male	85.0 (82.0, 89.0)	−0.078	0.938
	Female	85.0 (82.0, 88.0)		
The place of origin of the students	Rural	86.0 (84.0, 89.0)	−3.365	0.001
	Urban	85.0 (76.0, 87.0)		
Grade	Fourth year	86.0 (84.0, 89.0)	−1.605	0.109
	Fifth year	85.0 (79.5, 87.0)		
Students who have signed clinical work agreements	Yes	86.0 (82.5, 90.5)	−1.517	0.129
	No	85.0 (82.0, 87.3)		
The major of your college entrance examination is clinical major or not	Yes	85.0 (82.0, 88.0)	−0.833	0.405
	No	86.0 (84.0, 89.0)		
The degree of understand about general practitioners	Not really understand	89.0 (86.0, 99.5)	23.586	<0.001
	Don’t understand much	86.0 (83.0, 89.0)		
	General understand	85.0 (82.0, 87.3)		
	Better understand	85.0 (72.5, 86.0)		
	understand very well	62.0 (43.0, 84.0)		
internship time	1 week and under	85.0 (79.0, 88.5)	21.01	<0.001
	2 weeks	85.0 (82.0, 89.0)		
	4 weeks and above	86.0 (84.0, 89.0)		
What do you think of the salary and wages of general practioners	Very bad	89.0 (88.0, 94.0)	17.238	0.002
	Poor	86.0 (84.0, 91.5)		
	Average	85.0 (81.0, 87.0)		
	High	85.0 (83.0, 91.0)		
	Very high	128.5 (126.0, 131.0)		
How is your sense of identity for general practitioners?	Lower	83.5 (74.0, 87.0)	18.405	<0.001
	Low	85.0 (81.0, 89.0)		
	Average	85.0 (84.0, 88.0)		
	Higher	85.0 (88.0, 114.5)		
	Very high	79.0 (55.0, 86.5)		
What do you think of the employment prospects for general practitioners?	Very bad	89.0 (88.0, 110.0)	12.541	0.014
	Poor	85.0 (82.0, 91.0)		
	Average	85.0 (84.0, 87.0)		
	High	85.0 (80.5, 87.0)		
	Very high	89.0 (88.0, 110.0)		
Will you choose to become a general practitioner after graduation?	Yes	86.0 (84.0, 92.3)	−2.463	0.014
	No	85.0 (82.0, 87.0)		

**Table 13:** Evaluation table of factors influencing the general practice ability of medical undergraduates.

Factors	Variables	Assignment
General practice ability score	Y	0 = “≤85”; 1 = “>85”
Gender	X1	0 = “Male”; 1 = “Female”
The place of origin of the students	X2	0 = “Rural”; 1 = “Urban”
Are you an oriented student?	X3	0 = “Yes”; 1 = “No”
Whether clinical medicine is your first choice for higher education	X4	0 = “Yes”; 1 = “No”
The degree of understand about general practitioners	X5	0 = “low”; 1 = “high”
Internship time	X6	0 = “Under 2 weeks”; 1 = “2 weeks and over”
What do you think of the salary and wages of general practitioners	X7	0 = “Low”; 1 = “High”
How is your sense of identity for general practitioners?	X8	0 = “Low”; 1 = “High”
What do you think of the employment prospects for general practitioners?	X9	0 = “Poor”; 1 = “Good”
Will you choose to become a general practitioner after graduation?	X10	0 = “Yes”; 1 = “No”

**Table 14:** Logistic regression analysis of the influencing factors on the general practice ability of medical undergraduates.

Variables	B	SE	Wald	*p*	*OR (95%CI)*
The place of origin of the students	−1.056	0.311	11.499	0.001	0.403 (0.236–0.688)
Degree of understanding	−4.524	1.248	13.144	0.000	0.011 (0.001–0.125)
Internship time	1.118	0.371	9.084	0.003	3.058 (1.478–6.327)
Salary and wage	0.911	0.363	6.284	0.012	2.486 (1.220–5.068)
Professional identity	0.914	0.377	5.893	0.015	2.496 (1.193–5.222)

## Data Availability

The data presented in this study are available on request from the corresponding author.
